# Phanerozoic thermochronology record of Afro-Arabia through space and time

**DOI:** 10.1038/s41597-025-04767-6

**Published:** 2025-03-17

**Authors:** Samuel C. Boone, Malcolm McMillan, Maria-Laura Balestrieri, Barry Kohn, Andrew Gleadow, Abaz Alimanovic, Graham Hutchinson, Wayne Noble, Vhairi Mackintosh, Christian Seiler, Dave Belton, Danielle Majer-Kielbaska, Daniel F. Stockli, Joachim Jacobs, Edgardo J. Pujols, Matthias Daßinnes, Benjamin Emmel, Fabian Kohlmann, Romain Beucher

**Affiliations:** 1 University of Sydney, School of Geosciences, Camperdown, NSW 2050 Australia; 2 University of Melbourne, School of Geography, Earth and Atmospheric Sciences, Parkville, VIC 3010 Australia; 3 University of Adelaide, Department of Earth Sciences, The University of Adelaide, Adelaide, SA 5005 Australia; 4 Istituto di Geoscienze e Georisorse, Consiglio Nazionale delle Ricerche, UOS Firenze, Firenze, Italy; 5 Lithodat Pty Ltd, Melbourne, Victoria 3030 Australia; 6 Anglo American, 201 Charlotte Street, Brisbane, QLD 4000 Australia; 7 Department of Earth and Planetary Sciences, The University of Texas, Austin, Texas 78712 USA; 8 University of Bergen, Department of Earth Science, P.O. Box 7803, N-5020 Bergen, Norway; 9 Equinor ASA, EPN, 7502 Stjørdal, Norway; 10 SINTEF Industry, 7465 Trondheim, Norway; 11 Australian Earth-System Simulator National Research Infrastructure (ACCESS-NRI), Canberra, ACT 2601 Australia

**Keywords:** Geochemistry, Tectonics

## Abstract

Low-temperature thermochronology has been widely used in eastern Africa and Arabia (Afro-Arabia) to investigate the long-term thermal evolution of the crust in response to Phanerozoic tectonism. Yet, utilisation of this invaluable thermochronology record to inform numerical investigations into the long-term tectonothermal, geodynamic and landscape evolution of the region has been limited by the dispersion of these data across numerous disparate case studies. Here, we present a relational database of apatite (1787), zircon (68) and titanite fission-track (97) analyses, and apatite (1,945), zircon (3310), and titanite (U-Th)/He (83) ages, including 465 new fission-track and 2,583 new single-grain (U-Th)/He analyses from the region. Where available, all detailed data needed for performing thermal history modelling are presented. Also included are 668 digitised thermochronology-derived thermal history simulations. Collectively, this comprehensive database records the Phanerozoic thermal evolution of Afro-Arabia through space and time. The machine-readable database is made publicly available through the EarthBank platform, enabling 4D (3D through time) geospatial data interrogation.

## Background & Summary

**Fig. 1:**
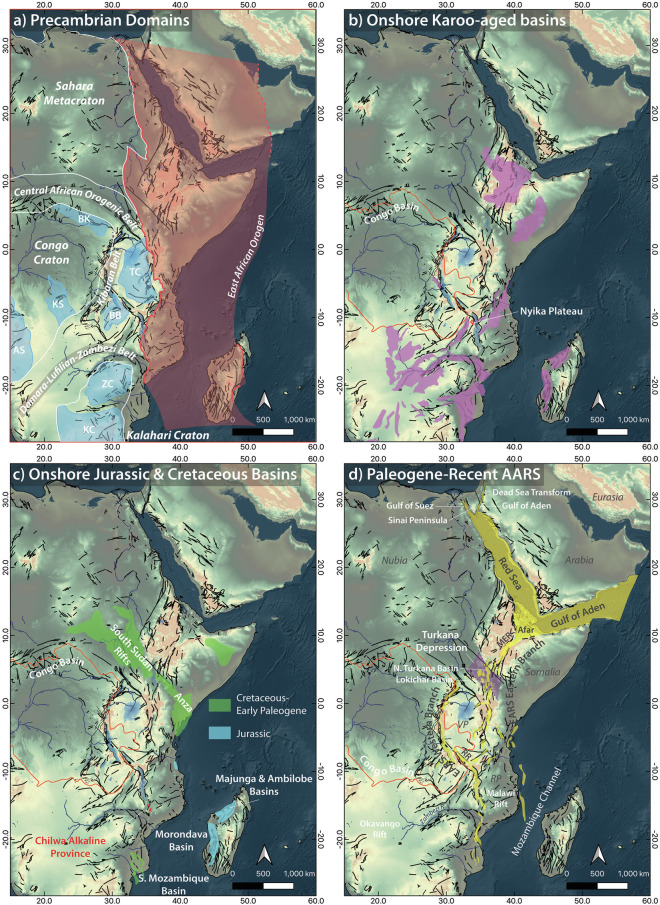
Key geological domains and features of eastern Africa and Arabia discussed in the text. Distribution of cratons, orogenic belts and basins after Purcell^174^, Davison and Steel^3^, Collins *et al*.^24^ and Celli *et al*.^175^. Fault map is from Thiéblemont *et al*.^176^. TC = Tanzanian Craton, BB = Bengweulu Block, BK = Bomu-Kibali Shield, KS = Kasai Shield, AS = Angolan Shield, ZC = Zimbabwe Craton, KC = Kaapvaal Craton.

Occupying the core of Gondwana since its formation in the late Neoproterozoic, eastern Africa and Arabia have experienced multiple periods of superimposed Phanerozoic extensional tectonism and related magmatism, periodically interrupted by spatiotemporally restricted compressional events, as the great southern hemisphere megacontinent slowly but inexorably broke apart (Fig. 1). Beginning with widespread extension throughout southern and eastern Africa during regional Permo-Triassic Karoo rifting^1^, the central Gondwanan lithosphere eventually began to rupture in the Jurassic as first Antarctica and then a still coupled Madagascar-India broke away from Africa and migrated to the south and east, respectively^2^. Widespread normal faulting, basin formation and subsidence continued through the Cretaceous and into the Paleogene along the passive East African margin and across much of northern Africa^3,4^.

This prolonged period of megacontinental disintegration eventually culminated in the development of the Eocene-recent Afro-Arabian Rift System (AARS), with extensional strain primarily accommodated along the three-pronged Red Sea, Gulf of Aden and East African Rift System (EARS). Nucleating first in the late Paleogene in discrete zones of extensional strain and magmatism in the Turkana Depression^5–8^, Afar^9,10^, and Rukwa rift sectors^11^ above a mantle superplume impinged beneath eastern Afro-Arabia^7^, the AARS has gradually propagated outwards to form a near continuously linked system of rift basins by the Quaternary^12^. As a result, the Nubian (African), Somalian (East African) and Arabian plates, along with the Victorian and Rovuma microplates of central Africa, have been kinematically decoupled^13^. This has enabled Arabia to independently migrate northeastwards since the Miocene leaving the newly formed oceanic lithosphere beneath the Gulf of Aden and Red Sea in its wake^14,15^. Meanwhile, the Somalian plate has rotated clockwise relative to Nubia as the East African Rift continues to accommodate ~E-W extension and normal faulting propagates southwards into the nascent rifts of the Okavango and Mozambique Channel^13,16^.

Pinpointing the spatiotemporal chronology of these tectonic events through deep time and thus the geodynamic systems governing these processes has, however, proved challenging. This is in part because the geological strata which directly record these phenomena in the deep past are often poorly preserved in outcrop, and publicly accessible subsurface data remain sparse. While geophysical techniques have provided a powerful toolset with which to assess the thermomechanical state of the Afro-Arabian lithosphere and underlying asthenosphere^17–19^, the snapshots which these data afford are restricted to the present-day and must be ‘reconstructed’ back through time to develop models of asthenospheric convection and lithospheric deformation processes operating in the deep past. These geodynamic simulations are themselves often very difficult to validate. Other geoscientists have turned to geochemistry as a tool for constraining the timing and rate of geological processes directly recorded in the chemical and isotopic composition of geological materials. This has included high-temperature geochronology, isotope geochemistry and major, minor and trace element geochemistry, which have been particularly important for tracking the magmatic history of the region^8,20–22^ and the metamorphic evolution of the Afro-Arabian crust^23–25^.

However, to constrain past lower-temperature geological processes which operated in the middle to upper crust, geoscientists have instead had to rely on so-called low-temperature thermochronology techniques. Applied primarily to outcropping Precambrian crystalline granitoids and metamorphic assemblages, and to a lesser degree Phanerozoic sedimentary rocks sampled at the surface or at depth in boreholes, these temperature-sensitive dating techniques have enabled geoscientists to directly constrain the thermal evolution of the Afro-Arabian upper crust in response to multiple, superimposed periods of orogenesis, rifting, magmatism, hydrothermal fluid flow, dynamic uplift, and long-term erosion. In particular, apatite fission track (AFT) and (U-Th)/He (AHe) analyses have been widely utilised in an effort to constrain the timing and rate of rift-related, upper crustal thermal perturbations between ~30 and 120 °C (up to ~5 km depth)^5,26–89^. In turn, these provide insights into the spatio-temporal evolution of individual rift basins^5,26,39,40,64,69,77,90,91^, development of rift-related topography^65,76,81^, normal fault system growth^60,66,92^, sediment provenance^87,93^, and, in some cases, the thermal influence of igneous intrusions and circulation of heated fluids^84^. In many cases, these data have been complemented by the utilisation of higher temperature thermochronometers, such as zircon and titanite fission track (ZFT and TFT, respectively) and (U-Th)/He (ZHe and THe, respectively) to investigate periods of mid-crustal exhumation related to older tectonic events which shaped the lithospheric architecture in which the AARS developed^33,34,38–40,42,43,54,61–63,65,71,76,80,82,84,94,95^, date igneous intrusions^51,73^, or as a tool for tracking source-to-sink sediment transport histories^87,93^.

Yet, the relatively limited number of samples and confined spatial extent of individual case studies have precluded insights into longer wavelength tectonic and geodynamic phenomena, such as regional denudation trends and the growth of topography due to plume impingement. This has spurred authors to begin synthesising low-temperature thermochronology data on regional scales in an effort to constrain the spatio-temporal evolution of entire rift segments, such as in the wider Red Sea and Gulf of Aden^90,96,97^. Yet, the raw data which underpin these regional syntheses remain buried in the many dozens of publications and associated appendices, requiring readers to manually mine these data if they wish to make use of them in their research.

**Fig. 2:**
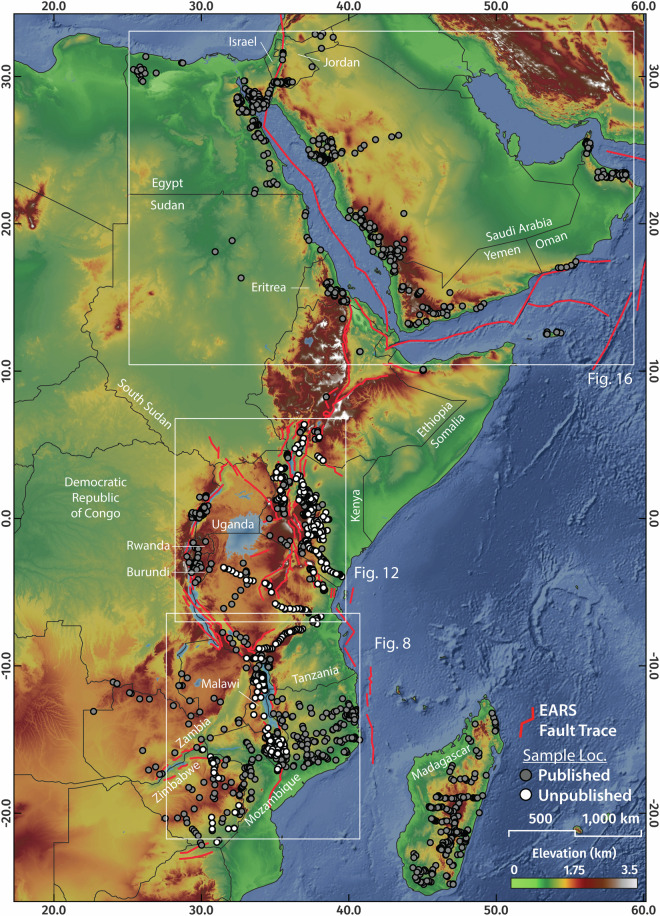
The greater Afro-Arabian Rift System region showing previously published and previously unpublished low-temperature thermochronology localities.

**Fig. 3:**
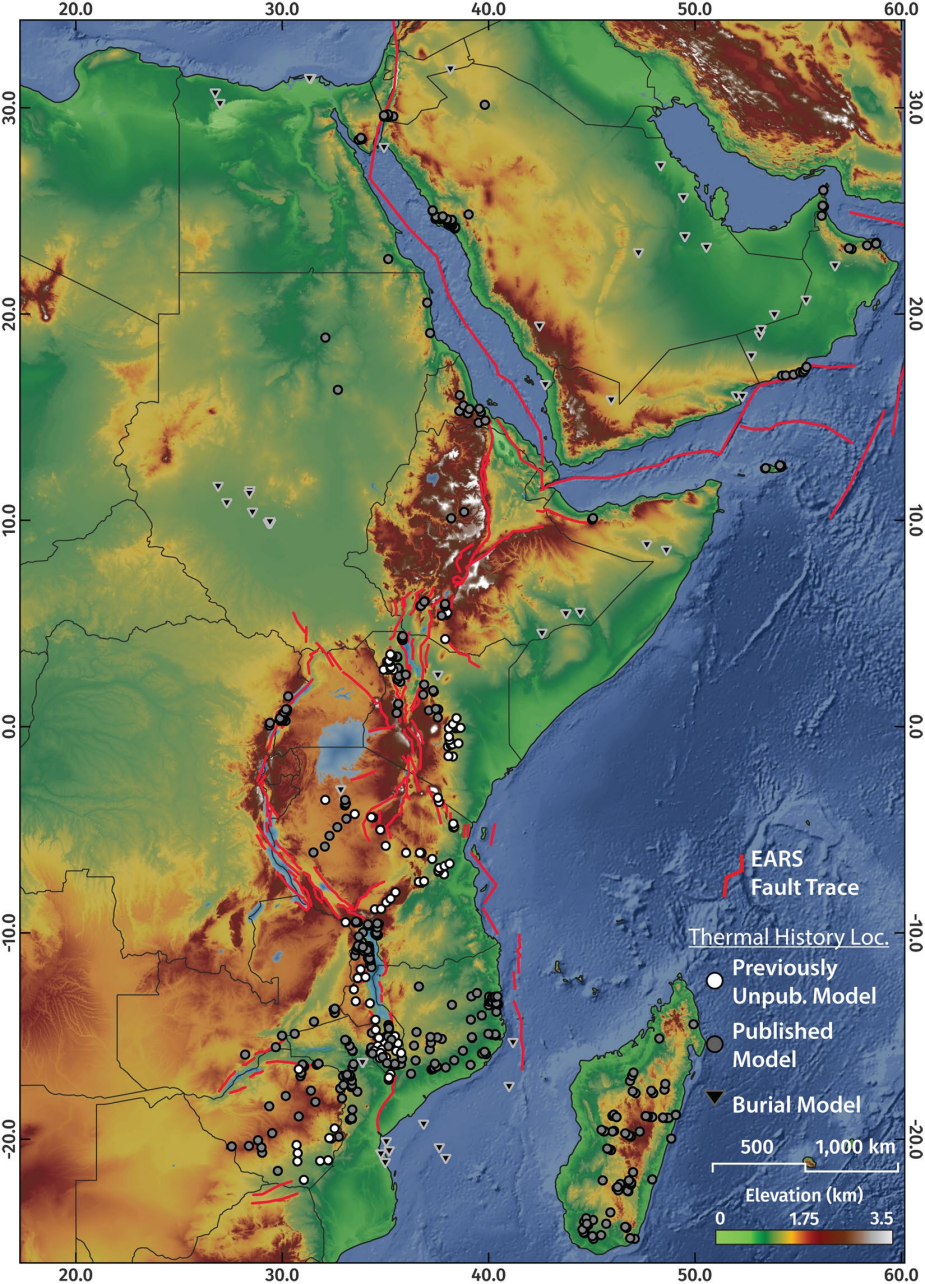
The greater Afro-Arabian Rift System region showing the locations of digitised published and previously unpublished thermal history simulations and burial history models.

Here, we present a synthesis of 1787 AFT, 68 ZFT, 97 TFT, 1,715 single grain and 230 multi-grain aliquot AHe, 3310 single-grain ZHe, and 61 single-grain and 22 multi-grain aliquot THe ages from across the greater Afro-Arabian Rift System region^98^, representing every known low-temperature thermochronology analyses to exist from the area as of March 2023 (Fig. 2). This comprehensive dataset includes 465 new fission-track and 2,583 new single-grain (U-Th)/He analyses from 833 rock samples from Egypt, Ethiopia, Kenya, Malawi, Mozambique, and Zimbabwe which are previously unpublished. Where available, we also present the associated electron probe microanalysis (EPMA) data that was collected in tandem with AFT analysis. The database also includes an additional 668 digitised thermochronology-derived thermal history models and burial history models from well data (Fig. 3), which quantify the Phanerozoic time-temperature paths of outcrop and near-surface rocks in these locations.

The entire database^98^ is made publicly available via EarthBank (formerly called AusGeochem; https://ausgeochem.auscope.org.au/)^99,100^, an open-access geospatial data platform which enables geochemical, geochronological and thermochronological analyses to be disseminated and interrogated in 4D (3D through time). In addition to the sheer number of analyses, the level of data granularity is also unprecedented in low-temperature thermochronology databases, with all available measured parameters and analytical metadata included to maximise the utility of this powerful dataset.

This continental-scale low-temperature thermochronology synthesis provides novel insights into the upper crustal evolution of the AARS that were previously difficult to decipher from an otherwise cumbersome and intractably large dataset. The data record a series of pronounced episodes of upper crustal cooling related to the development of the Red Sea, Gulf of Aden, and East African Rift System (EARS) since the late Paleogene. In addition, they provide insights into the inherited Phanerozoic tectono-thermal histories of these regions which controlled the spatial and temporal distribution of subsequent igneous activity and extensional strain. Taken together, this dataset provides an invaluable geochemical record of the Phanerozoic thermal evolution of East Africa and Arabia with which to validate future tectonic studies and numerical geodynamic and landscape evolution models of the region.

## Methods

Thermochronometers, such fission track and (U-Th)/He analysis, are temperature sensitive radiometric dating techniques where the retention of radiogenic daughter products in a mineral sample is a product of time, temperature, cooling rate and crystal chemistry^101,102^. To quantify the thermal histories which these data record, numerical thermal history modelling is often performed using programs such as QTQt^103^ and HeFTy^104^ and published fission-track annealing^105^ and He diffusion models^106–109^. The time-temperature histories of geological materials recorded by thermochronology can thus provide important constraints for the advection of mass and heat in the crust, which generally reflect processes such as tectonic exhumation, erosional denudation and subsidence, or in some instances, conductive heating due to changes in crustal heat flow, magmatism or hydrothermal activity^110–112^.

The titanite, zircon and apatite fission-track and (U-Th)/He database presented herein^98^ consists of a compilation of 4,227 published and 3,048 new, previously unpublished low-temperature thermochronology data from the greater Afro-Arabian Rift System region. For previously published work, detailed geosample and analytical (meta)data were mined from their respective publications, cleaned and compiled into the relational thermochronology data models of Boone *et al*.^100^, to enable more efficient reutilisation of these invaluable data by the scientific community. For descriptions of the methodologies employed to generate these data, readers should refer to their associated references, all of which are listed along with the data in the datafiles. The analytical methodologies for the newly published data are presented below.

The compilation also includes 668 digitised thermal history models, comprised of 70 published burial history models generated from well data^71,113–136^ and 598 thermochronology-derived thermal history models (111 previously unpublished), the metadata and resulting simulated time-temperature paths of which are included in this database.

### Thermochronology samples

**Fig. 4:**
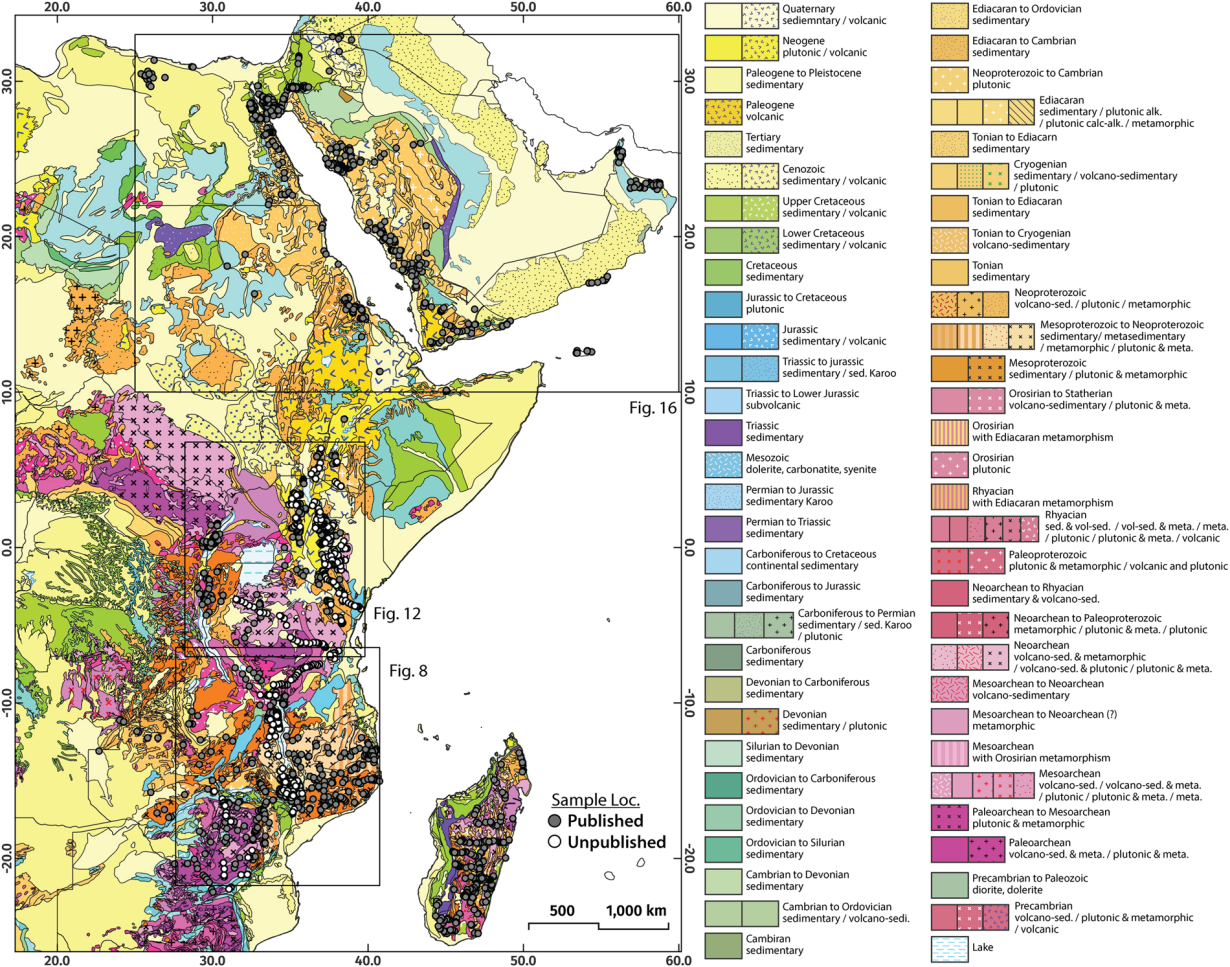
Simplified geological map of the greater AARS region showing the spatial distribution of thermochronology samples. Geological base map sourced from Thiéblemont *et al*.^176^.

**Fig. 5:**
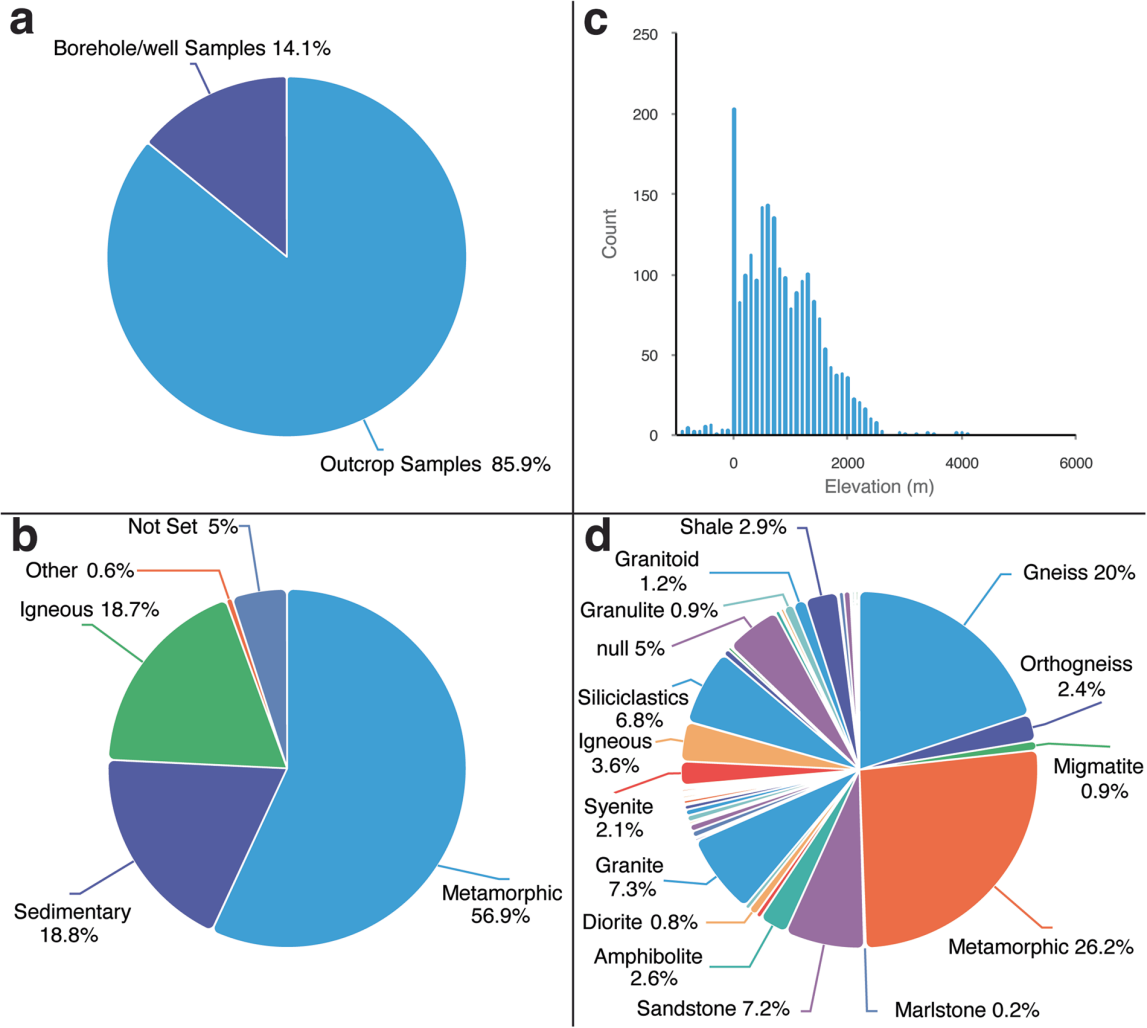
Geological sample metadata. Histogram and pie charts generated with EarthBank^99^ show the breakdown of sample types (**a**), elevations (**b**), rock types (**c**) and lithologies (**d**) in the database^98^ (n = 2449 geological samples).

The thermochronology data presented in this compilation^98^ were acquired from 2449 rock samples (primarily Precambrian metamorphic and igneous lithologies), 85.9% of which were collected from surface outcrops from elevations ranging from 0 to 4144 m above sea level (Figs. 4, 5). The remaining 14.1% were collected from boreholes and exploration wells down to depths of 936 m below the surface.

### Apatite fission track thermochronology

Fission-track analysis is based on the spontaneous fission of ^238^U and the resulting formation of crystallographic damage trails in a uranium-bearing mineral, called fission tracks^101,137,138^. These tracks anneal rapidly above a mineral-specific temperature threshold, while below a lower threshold, they retain nearly the full, original fission-track length. Within the intermediate partial annealing zone (PAZ), fission tracks anneal progressively as a function of time, temperature, cooling rate and chemistry^105,139^. In the apatite fission-track (AFT) system, the PAZ typically ranges from ~60 to ~110–120 °C for fluorapatite over geological timescales^140^, depending on mineral composition and heating duration^141,142^. Higher-temperature systems, such as titanite (TFT) and zircon (ZFT), have PAZ ranges of ~310–265 °C and ~350–200 °C, respectively^143–149^. By analyzing both fission-track ages and the distribution of confined track lengths—often summarized by mean track length (MTL) and standard deviation—the thermal history of a sample within the PAZ can be reconstructed^103,104^.

#### External detector method fission track analysis

Previously unpublished external detector method (EDM) fission track analyses presented herein were performed across two laboratories: the Istituto di Geoscienze e Georisorse, Consiglio Nazionale delle Ricerche, in Firenze, Italy and in the former home of the Melbourne Thermochronology Research Group at La Trobe University in Melbourne, Australia.

For all EDM analyses, apatite grains were mounted in epoxy resin, ground, and polished to expose internal mineral surfaces. Samples were etched with 5 M HNO_3_ at 20 °C for 20 s to reveal spontaneous fission tracks intersecting the polished internal apatite crystal surfaces. Samples were covered with a uranium-free muscovite external detector and irradiated with thermal neutrons; those analysed at the Istituto di Geoscienze e Georisorse were irradiated at the Laboratorio Energia Nucleare Applicata Triga Mark II reactor at Pavia University, Italy, while those analysed at La Trobe were irradiated at the Australian Institute of Nuclear Science and Technology Organisation HIFAR reactor. Induced fission tracks in the external detector were revealed by etching the mounts in 40% HF at room temperature for 40 min.

Fission track counting and length measurements of EDM samples from Ethiopia was performed by author Maria Laura Balestrieri on a Zeiss Axioskop (under a nominal dry objective magnification of 1250x) equipped with a Kinetek automatic stage at the CNR-IGG Fission-Track laboratory. The Trackkey 4.2 Program was used for all AFT age calculations procedures^150^ using a ζ-calibration of 360 ± 11.

Determination of fission track densities and the measurement of horizontal-confined track lengths for all remaining EDM samples were performed at La Trobe University by authors Wayne Noble (ζ-calibration of 354 ± 7) and Dave Belton (ζ-calibration of 362.4 ± 12) using an Autoscan TM stage system and a HIPAD TM digitizing tablet, in conjunction with a Zeiss microscope (at 1250x magnification) with dry objectives and a drawing tube.

For all EDM analyses, a chi-square (χ^2^) test was carried out on the AFT single-grain age in order to test homogeneity of data^151^. The probability of (χ^2^) was calculated for each sample: if P(χ^2^) > 5% then the sample is assumed to be comprised of a single population of single-grain ages^152^.

Track-pit opening diameters parallel to the crystallographic c-axis (Dpar) were measured for each grain as a proxy for the compositional control on fission track annealing kinetics^141^. Dpar lengths have been shown to correlate with various chemical compositions that affect annealing, such as Cl, F, and OH. In general, apatite grains with longer Dpars show increased resistance to annealing and are likely correlated with higher concentrations of Cl and OH with respect to F^153^.

#### Laser ablation inductively coupled plasma mass spectrometry fission track analysis

New laser ablation inductively coupled plasma mass spectrometry (LA-ICP-MS) fission track analyses were acquired at the University of Melbourne Thermochronology Laboratories in Melbourne, Australia following the analytical protocol of Seiler *et al*.^154^. Two apatite grain mounts were prepared for each sample (FT counting and FT length) using two-part cold-set epoxy on glass slides. Both slides were polished to a 1 μm finish on a Struers Rotopol automated polishing machine using diamond pastes. Slides were etched in 5 M HNO3 at 20 °C for 20 s.

Analyses were performed on stacked image sets from the grain surface to a depth of ∼15 μm in reflected and transmitted light. The *TrackWorks* was used to automatically capture digital images using a 4.0 MP IDS uEye UI-3370CP-C-HQ camera mounted on a Zeiss M1m AxioImager microscope with a 1,000x total magnification and a 100x dry objective (calibration = 0.0873/0.0873 μm/pixel). On average, 30 suitable grains were selected for counting spontaneous fission track density measurements, which were obatined autonomously by *FastTracks* using the “coincidence mapping” technique of Gleadow *et al*.^155^ and manually corrected where necessary.

Single grain uranium concentrations were determined by LA-ICP-MS single spot analysis using a New Wave Nd:YAG Laser (λ = 213 nm with 5 Hz @ 56%–57% power, spot size = 30 μm, ∼8 μm ablation depth) carried with Ar gas to an Agilent 7700 mass spectrometer. The well-characterized Durango apatite and an in-house sintered Mud Tank carbonatite apatite were used as secondary reference materials for LA-ICP-MS data quality assessment. Single grain and pooled ages were calculated according to Seiler *et al*.^154^ using an ξ-calibration of 2.185 ± 0.134 × 10^−3^. Central ages were calculated following Vermeesch^156^, recasting 0-track grains to an EDM-like form.

Confined track lengths (Track-In-Track [TINT] only) were measured as true-dip corrected 3D lengths up to 20° using *FastTracks* after irradiation by ^252^Cf (to increase TINT occurrence) and corrected for a 1.634 refractive index (apatite).

### Electron probe microanalysis

Numerous compositional factors, particularly cation substitutions, have been shown to influence fission track annealing in apatite^142,157^, highlighting the limitations of Dpar as a standalone proxy for grain composition. Therefore, in addition to measuring Dpar, individual apatite crystal compositions were directly analyzed for all LA-ICP-MS fission track samples using electron probe microanalysis (EPMA). A JEOL JXA-8530F FEG electron probe microanalyser was used with five WDS channels and an SDD EDS detector, operating at 15 kV accelerating voltage, 10 nA beam current, and a 10 μm beam diameter. Standard LA-ICP-MS fission track analyses included measurements of F, P, Cl, and Ca. For the most recent LA-ICP-MS fission track results from the Malawi Rift region, additional EPMA measurements were obtained for Sr, Na, Ce, La, Mn, Mg, Fe, K, Y, S, and Si. These data were used to assess annealing resistance via the multi-compositional model of Ketcham *et al*.^105^, employing rmr_0_ as a proxy (Eq. 11; Ketcham *et al*.^105^).

### Zircon and apatite (U-Th)/He thermochronology

(U-Th)/He thermochronology is based on the production of ^4^He during the radioactive decay of ^238^U, ^235^U, ^232^Th and, to a lesser extent, ^147^Sm. The contribution of ^4^He from ^147^Sm decay is typically < 1% of total radiogenic helium and is often negligible in U- and Th-rich minerals like zircon. However, in U- and Th-poor minerals such as apatite, the contribution of ^4^He from ^147^Sm decay must be considered. ^4^He diffusivity depends primarily on time, temperature, crystal size, and accumulated radiation damage^107,158–160^, though additional crystallographic and compositional factors can cause significant age dispersion (see Wildman *et al*.^161^; Danišík *et al*.^162^). In apatite, helium diffusion accelerates above ~40 °C and occurs almost instantaneously above ~80 °C^163^, defining the apatite (U-Th-Sm)/He (AHe) partial retention zone (PRZ). The zircon (U-Th)/He (ZHe) system is sensitive to higher temperatures, with the ZHe PRZ typically spanning ~130–200 °C^164–166^, though grains with high radiation damage may exhibit lower closure temperatures^107^. The titanite (U-Th)/He (THe) PRZ ranges between ~180 and 100 °C^167^.

Previously unpublished apatite and zircon (U-Th)/He analyses presented here were obtained in the University of Melbourne Thermochronology Laboratories. The analytical procedure outlined in Gleadow *et al*.^168^ were employed (except that for ZHe analysis, ^233^U and ^229^Th spikes were used), with Durango apatites and Fish Canyon Tuff zircons being used as internal standards.

Clear, euhedral, and non-fractured apatite and zircon grains were manually selected under an Olympus SZX12 binocular microscope and measured for α-ejection correction following Farley *et al*.^169^. Grains were then placed in ethanol and examined under polarized light to check for inclusions. Apatite grains, sealed in acid-treated platinum capsules, were outgassed under vacuum at ~900 °C for 5 minutes using a Coherent Quattro FAP 820-nm diode laser with fibre-optic coupling. Zircons were outgassed at ~1300 °C using ~12.6 W laser power for 20 minutes to ensure complete ^4^He extraction. A hot blank was run after each gas extraction to confirm full outgassing, with second re-extractions contributing <0.5% of the total measured ^4^He across all samples. Helium content was determined by isotope dilution using a pure ^3^He spike, analysed on a Balzers Prisma QMS 200 quadrupole mass spectrometer and calibrated against an independent ^4^He standard.

Following outgassing, grains were removed from the laser chamber, dissolved, and analysed for parent isotopes using an Agilent 7700X ICP-MS. Apatite samples were digested in their capsules with HNO_3_ for ^238^U, ^235^U, ^232^Th, and ^147^Sm analysis. AHe analyses were calibrated using the BHVO-1 reference material, with Mud Tank Carbonatite apatite and the international rock standard BCR-2 as secondary references for each sample batch. AHe ages were calculated and corrected for α-emission following Farley *et al*.^169^.

Zircon grains were extracted from their Pt capsules, transferred to Parr bombs, spiked with ^235^U and ^230^Th, and digested in small volumes (0.3–0.5 ml) of HF at 240 °C for 40 hours. Standard solutions with identical spike amounts and unspiked reagent blanks were processed in the same manner. A second digestion in HCl at 200 °C for 24 hours ensured the complete dissolution of fluoride salts. The resulting zircon solutions were then dried down, dissolved in HNO_3_, and diluted in H_2_O to 5% acidity for ^238^U, ^235^U, and ^232^Th analysis via solution ICP-MS.

Analytical uncertainties, including α-ejection correction, an estimated 5-μm uncertainty in grain dimensions, gas analysis (<1%), and ICP-MS analytical precision, are conservatively estimated at ~6.2%. U, Th, and Sm content accuracy and precision typically fall below 1%, with a maximum uncertainty of ~2%.

### Thermal history and burial history models

Numerical thermal history models provide invaluable information about temporal, as well as spatial trends in upper crustal thermal histories. Only by time stamping periods of crustal thermal flux recorded by these data can the rates of geological thermal processes be determined and their potential temporal correspondence to other geodynamic and tectonic events be established. This also enables the use of these data as parameters or validation datasets for numerical geodynamic and landscape evolution models, which can simulate the thermal or exhumation history of the crust over geological timescales.

Thus, published thermal history models were also digitised where available, and archived in the relational data model format of Boone *et al*.^100^. Using the open-access WebPlotDigitizer software (version 4.2), best-fit, upper and lower 95% confidence envelope thermal history paths were extracted from published time-temperature plots. In addition, metadata pertaining to the modelling parameters and simulation goodness of fit were collated, enabling independent model quality assessment.

For 111 of the newly analysed samples, such as for previously unpublished thermochronology data from northwest Kenya, new thermal history modelling was performed to quantify the thermal evolutions which these data record. For previously unpublished data in Kenya, Ethiopia, Malawi and Zimbabwe, joint inverse numerical thermal history modelling of ZHe, AHe and AFT data was performed using QTQt^103^, using the multi-compositional AFT annealing model of Ketcham *et al*.^105^, the ZHe radiation damage accumulation and annealing model of Guenthner *et al*.^107^, and the AHe diffusion model of Gautheron *et al*.^108^. Only 1- and 2-termination ZHe and AHe grains were modelled, due to the tendency of 0-termination grains to yield significantly larger age dispersion^170^. A present-day surface temperature constraint of 20 ± 10 °C was applied to all models. The time-temperature modelling space was set as the oldest thermochronology age ± the oldest thermochronology age for that sample, along with the temperature range of 100 ± 100 °C. Thermal history modelling was first performed using AFT data only, before adding ZHe and AHe data in subsequent model runs. In two instances (samples TUB13-74 and TUB15-07), inverse models that included ZHe data predicted significantly different pre-middle Paleogene thermal histories than both inverse models of proximal samples expected to share a similar thermal evolution (TUB15-05 and TUB15-06) and models from the same samples that excluded ZHe data. These samples were then rerun with an additional time-temperature constraint between 70–50 Ma to test if the ZHe data from these samples were consistent with a similar period of Paleogene reheating predicted by their corresponding AFT data and the inverse models of neighbouring samples. Occasionally, the applied diffusion models were unable to reproduce the spread in observed ZHe or AHe data. In those cases, outputs from model runs that excluded those unreproduced data were preferred. Thermal history models of previously unpublished AFT data from Tanzania, by contrast, were generated using the MonteTrax software as described by Gallagher^171^.

While in a few instances thermochronology data from outcropping crystalline basement rocks record periods of reheating in response to burial^39,40,65^, thermochronology systems are in general more sensitive to cooling events^101,102^. Therefore, to address the inherent bias of the thermal history model compilation towards preferentially recording crustal cooling, the dataset also includes published burial history models generated from well data (Fig. 3). These data, by contrast, predominantly record periods of upper crustal heating during subsidence, sedimentation and heating.

**Fig. 6:**
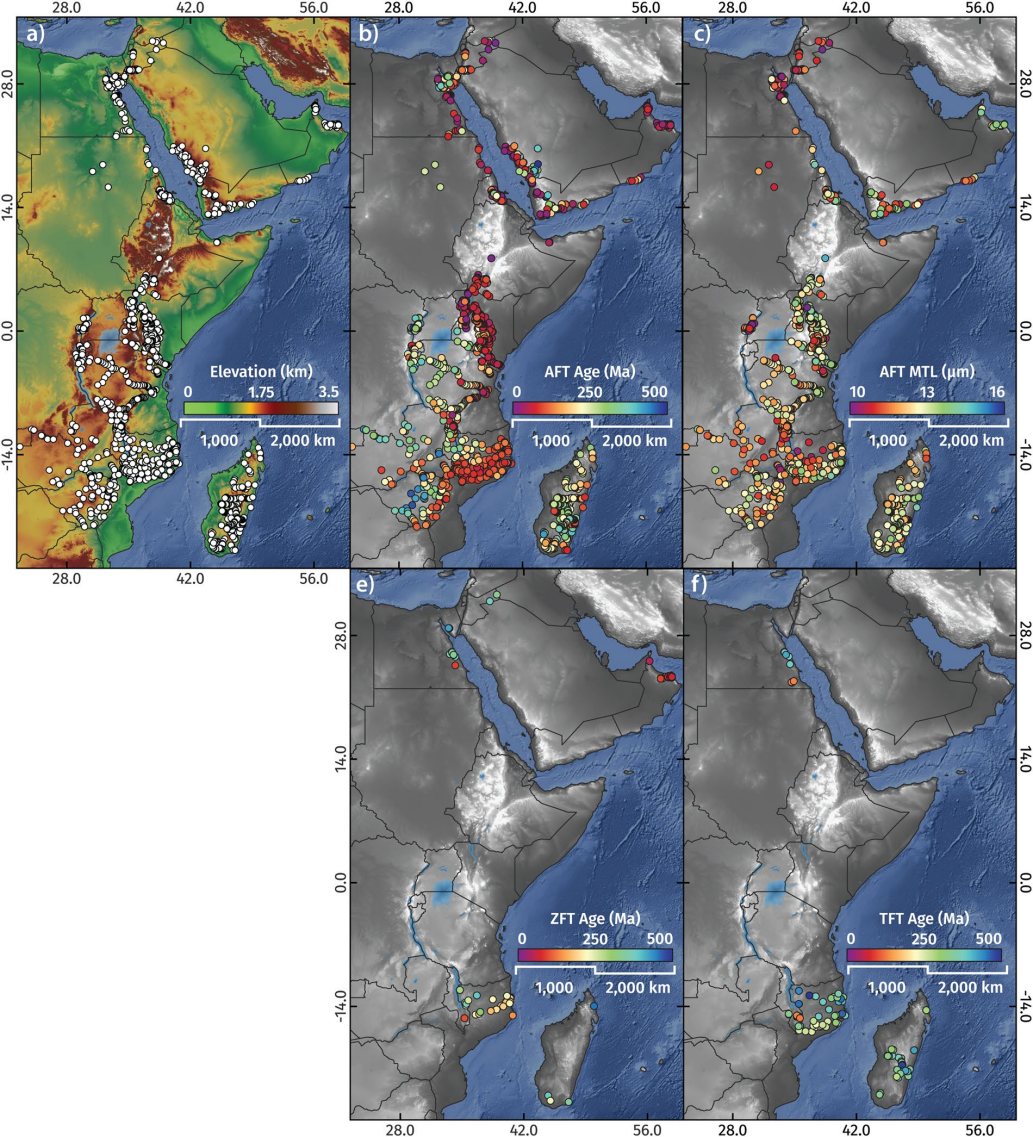
Fission-track data localities (**a**), apatite fission-track ages (**b**) and mean confined track lengths (**c**), zircon fission-track ages (**d**) and titanite fission-track ages (**e**) in the greater AARS region.

**Fig. 7:**
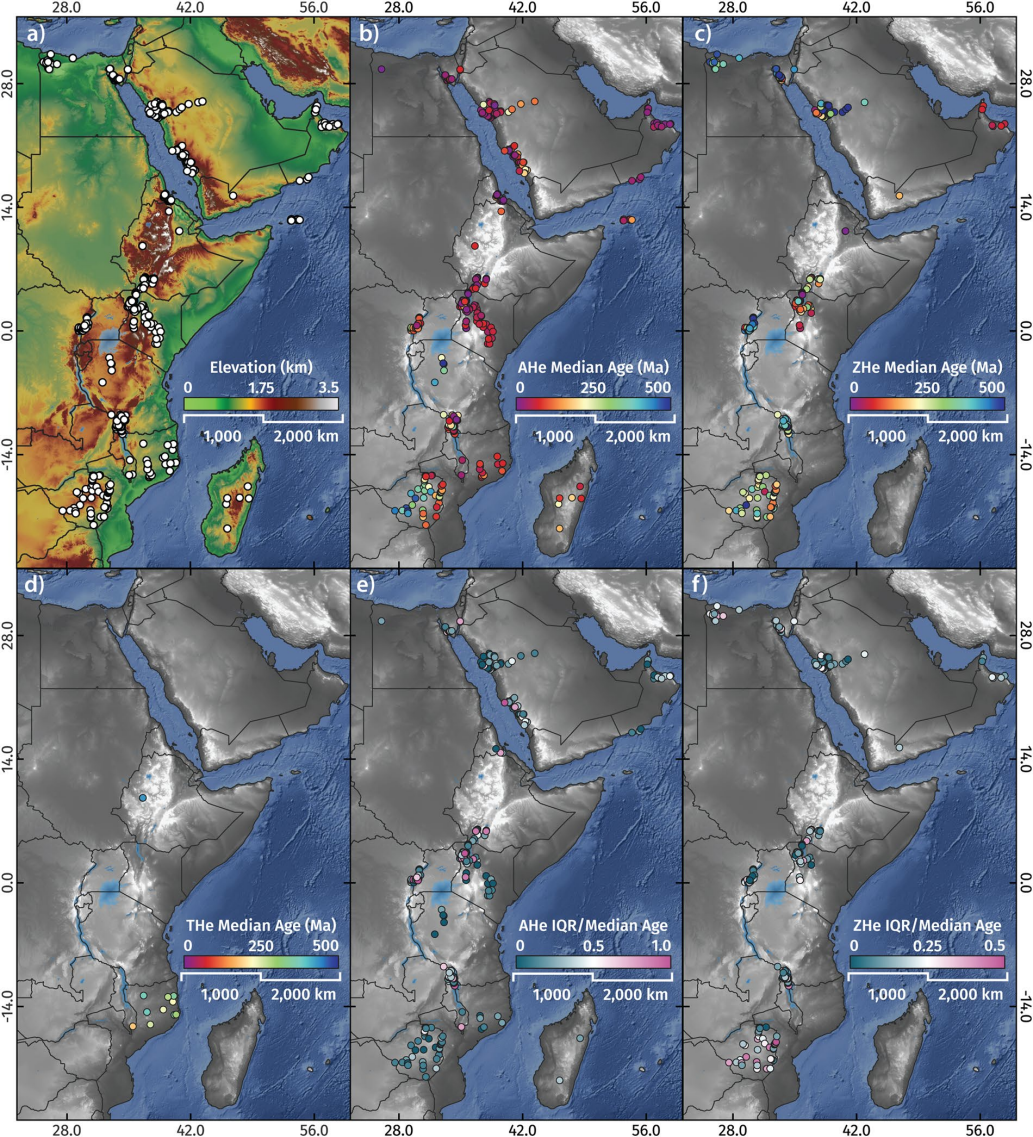
(U-Th)/He data localities (**a**), median apatite (U-Th)/He ages (**b**), median zircon (U-Th)/He ages (**c**), and median titanite (U-Th)/He ages (**d**) in the greater AARS region. Relative dispersion of apatite and zircon (U-Th)/He datasets are illustrated in (**e,f**), respectively, as single grain (U-Th)/He interquartile ranges divided by median ages.

The resulting collation presented here^98^ (Figs. 6, 7), which combines thermal history inversions of regional thermochronology data and burial history models of well data (locations of these models shown in Fig. 3), reveals spatiotemporal trends in Afro-Arabia crustal thermal evolution. These are recorded as paleotemperatures and cooling rates on a per-million-year basis that generally reflect periods of diachronous exhumation and burial in response to nearly 500 million years of tectonism.

## Data Records

The AARS thermochronology database^98^ is available for download from the EarthBank data platform^99,100^ (https://ausgeochem.auscope.org.au), itself licensed under a Creative Commons Attribution CC-BY 4.0 International License (10.58024/AGUM97FC4439).

The dataset includes detailed (meta-)data for geological rock samples and their associated fission-track and (U-Th)/He data. The geological rock sample metadata is reported following the vocabulary and data structure of Boone *et al*.^99^, including detailed location, information, sampling method, and lithological information, as well as the associated references for previously published analyses. The fission-track and (U-Th)/He data are archived in the relational database structure of Boone *et al*.^100^, itself built on the recommendations of the Geological Society of America Bulletin special edition on “Reporting and Interpretation of Chronology Data”^101,102^.

Data granularity varies drastically across the data compilation, with many legacy publications unfortunately only presenting the most basic geosample metadata and most cursory analytical data (e.g., sample location, age, and uncertainty). However, where previously published data were reported in high detail or for new fission-track data presented here, the records include detailed whole sample and single grain ages and chemistry, and confined track data on the per-track or binned (length histogram) basis. Wherever possible, including for all new analyses, (U-Th)/He data were reported in detail on a single-grain basis. Where previously published or for all new data, calibration factors and/or associated secondary reference material results are also included in the data compilation to enable independent data quality assessment.

## Technical Validation

Quality control of new thermochronological and geochemical analyses presented here was performed either via empirical calibration against primary reference materials or via the analysis of secondary reference materials of known age or composition, depending on the technique.

For apatite fission track analysis using the external detector method, an analyst-specific zeta factor was used to calibrate both the collection of difficult-to-measure variables (e.g., neutron fluxes) in the fission-track age equation and the counting behaviour of the analyst against the Durango and Fish Canyon Tuff apatite fission-track age standards (Hurford and Green, 1983). The zeta-factor for each external detector method apatite fission-track age determination is recorded in the data tables.

For laser ablation ICP-MS fission-track analyses, apatite ^238^U determinations were validated via parallel chemical measurements of the well-characterized Durango apatite (12.2 ± 0.1 ppm) and an in-house sintered Mud Tank carbonatite apatite (3.00 ± 0.01 ppm) as secondary reference materials, following the procedure of Seiler *et al*.^154^.

Prior to the EPMA of unknowns, JEOL JXA-8530F FEG electron probe microanalyser was tuned using a series of in-house standards of known composition for each element measured (F, P, Cl, Ca, Sr, Na, Ce, La, Mn, Mg, Fe, K, Y, S, and Si). The quality of each spot analysis was then assessed by their corresponding total count, with analyses yielding a count of 99–100.5 considered acceptable.

(U-Th)/He data quality was assessed via the analysis of secondary reference materials of known ages, the results of which are included in the data reports. For zircon (U-Th)/He, each analytical batch included multiple aliquots of Fish Canyon zircon (28.4 ± 0.2 Ma)^168^, while Durango apatite (31.02 ± 1.01 Ma)^172^ was run as a secondary reference material during apatite (U-Th-Sm)/He analysis. (U-Th)/He analyses of secondary reference materials consistently produced ages within error of the accepted values, yielding mean ages (reported at 2σ) for the Fish Canyon Tuff zircon of 28.6 ± 2.2 Ma (n = 9) and the Durango apatite of 31.1 ± 1.6 Ma (n = 25).

## Usage Notes

The low-temperature thermochronology database of the greater Afro-Arabian Rift System presented here^98^ can be freely accessed via EarthBank (previously AusGeochem^99,100^, https://ausgeochem.auscope.org.au/), a web-hosted, open-access geospatial data platform which enables researchers from around the world to archive, disseminate, and analyse geological, geochemical and geochronological/thermochronological data.

While the number of analyses included in this database may be large, the distribution of thermochronology data is uneven. The extent of available thermochronology data is spatially limited by surface geology and, in some cases, political boundaries. Thermochronology samples are almost exclusively limited to surface outcrops of Neoproterozoic crystalline basement and metamorphic rocks (Figs. 4, 5) due to challenges in untangling the mixed pre- and post-depositional thermal history recorded by many Phanerozoic sedimentary rocks. Moreover, a significant portion of thermochronology studies in the region have been focused on addressing questions pertaining to the late Paleogene-recent rifting, and thus data localities are disproportionally congregated around AARS basins. Consequently, the heterogenous spread in thermochronology data across the Afro-Arabian landscape has in many cases hindered addressing questions about earlier periods of Phanerozoic tectonics or the development of long-wavelength dynamic topography^173^. Elsewhere, there remains a complete lack of low-temperature thermochronology data, including along large swathes of the Red Sea, Gulf of Aden and East African coastlines, inhibiting more holistic studies of passive margin evolution that are not focused exclusively on areas with pronounced coastal escarpments.

Future studies in the region might then consider filling some of these data gaps with the aim of achieving a more homogenously distributed thermochronology record of the Phanerozoic Afro-Arabian upper crustal thermal evolution.

In places significant uncertainty remains as to the accuracy and geological significance of observed periods of cooling or heating, particularly further back in deep time where data coverage is poor. These issues, in part, may stem from differences in thermal history modelling codes, methodologies and parameters used to generate the array of published time-temperature reconstructions, and by thermal history simulation non-uniqueness. To overcome these discrepancies, future studies could consider utilising the Afro-Arabian thermochronology database to bulk model all samples, where detailed single grain ages, confined track lengths and kinetic parameter data exist, using a consistent modelling protocol.

Nevertheless, challenges in discerning the geological significance of specific periods of Afro-Arabian crustal thermal flux recorded by thermochronology data will remain. Disentangling these complexities will likely require future coupled geodynamic and landscape evolution modelling work that can account for 4D (3D + time) changes in crustal thermal regime due to the interplay of mantle processes, magmatism, brittle deformation, paleoclimate and surface processes.

## Data Availability

No custom was used in preparing this dataset or manuscript.
